# Factor Analysis of the Caregiver Quality of Life Index-Cancer (CQOLC) Scale for Chinese Cancer Caregivers: A Preliminary Reliability and Validity Study of the CQOLC-Chinese Version

**DOI:** 10.1371/journal.pone.0116438

**Published:** 2015-02-09

**Authors:** Jiaobo Duan, Jufang Fu, Hongjie Gao, Changsheng Chen, Jianfang Fu, Xin Shi, Xufeng Liu

**Affiliations:** 1 School of Psychology, Fourth Military Medical University, Xi’an Shaanxi, China; 2 Infection Control Center of Xijing Hospital, Fourth Military Medical University, Xi’an Shaanxi, China; 3 Department of Health Services, School of preventive medicine, Fourth Military Medical University, Xi’an Shaanxi, China; 4 Department of Statistics, School of preventive medicine, Fourth Military Medical University, Xi’an Shaanxi, China; 5 Endocrinology Department of Xijing Hospital, Fourth Military Medical University, Xi’an Shaanxi, China; 6 School of Pharmacy, Fourth Military Medical University, Xi’an Shaanxi, China; Iranian Institute for Health Sciences Research, ACECR, IRAN, ISLAMIC REPUBLIC OF

## Abstract

The English version of the Caregiver Quality of Life Index-Cancer (CQOLC) was translated into simplified Chinese (CQOLC-C), following cultural translation, back-translation and pretest steps. Three hundred and sixty one cancer caregivers participated in this study. Cronbach’s alpha was used to assess CQOLC-C reliability. Exploratory factor analyses (EFA) was used to generate two models of the measure’s factor structure, and confirmatory factor analyses (CFA) were used to test each model, such that the best model to explain the latent structure of the CQOLC-C was identified. EFA using different factor extraction methods yielded two models including four and eight factors. According to the CFA results, model 2 was better fit for the original study data, based on the RMSEA criterion [0.058(90% CI = 0.051-0.065)], χ^2^ (531) = 853.92, p < 0.0001; CFI (0.96), NNFI (0.96), IFI (0.97), and NFI (0.92). We also examined the effect of removing three items on the CQOLC-C factor structure and discuss the resulting differences from other versions. These results indicate that the CQOLC-C’s factor structure does not fully fit the original theorized model. This study provides preliminary support for further use of the CQOLC-C. However, the present work provides only partial support for the relevance and construct validity of the scale for Chinese caregivers.

## Introduction

Cancer is a growing problem world-wide. For several decades, more humans have been dying annually from cancer than from HIV/AIDS, tuberculosis and malaria combined [1]. In China, 1.96 million have died from cancer each year, a figure that constitutes a quarter of cancer deaths worldwide since 2008. Among all life-threatening illnesses, cancer is now the number one cause of death in China [2].

Cancer is a stressor that affects patients and their spouses, parents, adult children, and other family members who often serve as unpaid, informal caregivers [3]. Both patients and caregivers have been shown to experience similar levels of psychological distress during the early survivorship phase as well as years after diagnosis [4]. Often under tremendous pressure, caregivers are nevertheless required to meet the patient’s many needs, including treatment monitoring, treatment-related symptom management, emotional, financial, and spiritual support, and assistance with personal and instrumental care [5]. The impact of these overwhelming tasks can be considerable and over 30% of such caregivers live with clinically significant psychological distress and suffer poor quality of life (QoL) [6].

QoL of family caregivers of patients with terminal illness has been extensively studied in Europe and America over the past decades. Caregiving exerts great influence on caregiver QoL. According to previous studies, caregiver QoL is multidimensional [7,8,9] and includes psychological, social, mental, physical, spiritual, and behavioral components, not only during the time that they are providing care but throughout the trajectory of the illness [10]. However, QoL of cancer patient caregivers has rarely been studied in the Asia Pacific Zone. This gap may be due to lack of a proper assessment instrument [25].

While there have been many instruments developed to measure cancer patients’ QoL—in 1996 there were more than 21 instruments in the cancer field alone [11]—before 2003 only three instruments were available that specifically measure caregiver QoL [12,13,14]. The Quality-of-Life Index-Cancer (CQOLC) is a multidimensional tool [15], with this instrument showing particular promise in terms of capturing most theoretical dimensions of caregiver QoL [16, 17]. The CQOLC was developed by Weitzner in 1997 [18]. It was rigorously developed over a series of phases, using new groups of cancer patients and their caregivers at each developmental stage. In a succession of published articles, the authors/developers described these steps as well as use of the instrument to compare caregivers of patients in a curative versus palliative care setting [18, 19]. The instrument’s psychometric qualities were comprehensively tested using proper statistical tests and comparisons with other established instruments. In addition, the reliability and validity of the CQOLC have been demonstrated in the United States [18–20] Korea (CQOLC-K) [21], Turkey (CQOLC-T) [22, 23] and France (CQOLC-F) [24]. There is also a Mandarin version (Traditional Chinese) (CQOLC-M), tested using a Taiwanese sample [25]. To the best of our knowledge, the CQOLC has not been studied on the Chinese mainland. Unfortunately, no other QoL instrument exists for use in this region. Due to cultural and language differences between Taiwan and the Chinese mainland, it is vital and necessary to develop a simplified Chinese (Putonghua) version of the CQOLC (CQOLC-C). We translated the original CQOLC into simplified Chinese and aimed to provide preliminary support for use of the resulting CQOLC-C in Chinese cancer medical practice and cross-cultural cancer research.

## Materials and Method

### Participations and procedure

A total of 370 caregivers from 5 hospitals in Xi’an city participated in this study. The inclusion criteria for family caregivers were as follows: (1) a relative of cancer patient, (2) identified by the patients as the unpaid persons most involved with their actual care, (3) 18 years of age or older, and (4) willing to participate and able to communicate with the researchers. Nine caregivers did not complete the questionnaire due to physical problems or treatment matters (such as the caregiver having to take the patient to an examination on the date the questionnaire was to be completed). Analyses were conducted on data provided by 361 caregivers who completed the questionnaire. There were no missing data points.

According to previous studies, CQOLC factor structures vary across versions [18–25]. The purpose of this study was to assess the validity of the CQOLC-C by performing both exploratory factor analysis (EFA) and confirmatory factor analysis (CFA), both excellent methods to evaluate the latent structure of a scale.

### Caregiver Quality of Life Index-Cancer (CQOLC)

The CQOLC is a self-administered scale specifically designed to evaluate cancer patient caregiver QoL. This scale includes 35 items. CQOLC responses are scored from 0 (not at all) to 4 (very much). The total possible score is 140, with higher scores representing better QOL. In the original study, evidence supported four subscales: Burden, disruptiveness, positive adaptation, and financial concern [20]. These four factors include 27 items, with 8 additional items not loading onto these factors. Adequate internal consistency has been demonstrated, with internal consistency values (Cronbach’s alpha) for the four subscales being 0.89, 0.83, 0.73, and 0.81, with a value of 0.90 for total CQOLC scores.

The development of the CQOLC-C proceeded according to steps followed in previous cross-cultural studies [26, 27]. The process included cultural translation of items, back translation of items, and a pretest. Initially, two native Chinese speakers (proficient in English) performed the cultural translation, replacing words or phrases in English with phrases that are appropriate for Chinese culture. For example, the word “die” in item 9 was changed to “pass away”. This step resulted in another English language version, E1. The second step was back translation. The two experts translated the E1 version into simplified Chinese, the C1 version. Two other Chinese experts (who lived in English speaking countries for two years or longer) then translated C1 back into English to yield E2. Next, a native English speaker compared E2 to the original English version, providing opinions. The language experts then refined the scale and took the pretest. These steps have been repeated four times.

### Ethical consideration

Permission to conduct this study was granted by the authors’ institutional ethical committee. Informed consent was obtained from the Director of the Hospitals and the head of the clinical unit as well as all study participants. Cancer caregivers were informed about the purpose of the study and what would be expected of them. Participants were assured of their rights of refusal to participate in or to withdraw from the study at any stage without negative consequences. Anonymity and confidentiality of participants were guaranteed.

## Data Analysis

All items were coded and scored, and all data were entered, checked for missing values, and analyzed using SPSS version 18.0 and Lisrel 8.70 statistical software. There were no incomplete questionnaires or missing data. In order to ensure accuracy, we required adequate sample sizes, both in terms of absolute numbers and subject-to item ratios (5-10 subjects per item) [28]. There are 35 items in CQOLC, in this case, the sample size of this research should be 175-350 (35 items). We randomly selected 180 individuals from the total sample of 361 to provide data for EFA, with the remainder providing data for the CFA. Descriptive statistics (means and standard deviations) are reported for the main variables.

### Exploratory factor analysis

The initial stage of our analysis sought to establish the latent structure of the CQOLC-C, using EFA to identify a viable factor structure for the 35 items. A principal component extraction method with varimax rotation was used. Considering cultural differences between China and the United States and studies of previous versions, we set two models. M1 follows the results of the original study, wherein the factor structure was limited to four factors. In M2, factors with eigenvalues greater than 1.0 were identified for factor retention. Items with factor loadings (FLs) greater than 0.40 (including values that rounded to 0.40) and that did not load on more than one factor were retained. Items not meeting these criteria were removed one at a time.

### Confirmatory factor analysis

Confirmatory factor analysis is used to determine goodness of fit between a model already obtained by another researcher and the present sample data.[29] A CFA with the normal theory maximum likelihood estimation method was conducted on our sample to confirm the two exploratory models. Several goodness-of-fit indices were computed to assess degree of fit. A goodness-of-fit index (GFI) of at least 0.90 has been the standard adopted by many researchers. The criteria were the same as those used in the study of Zeynep, C. O and Bektas H.A. [23].

## Results

Caregivers were, on average (SD), 40 years old (12.17). Most were between 31 and 45 years old (n = 134, 37.1%). Most were women (n = 194, 53.7%). Most caregivers were married (n = 291, 80.6%), and most of them (n = 202, 56.0%) were employed. Their income levels were below and equal to the average wage (n = 179, 49.6%, n = 172, 47.6%). Similar to a finding from Taiwanese research [25], the largest proportion were spouses (n = 131, 36.3%).

According to the criteria outlined above, there are four latent factors (subscales) in M1 and eight factors in M2. These two models were both rotated by varimax, accounting for 45.885% and 64.516% of the total variance (Tables 1 and 2).

**Table 1:** EFA of 4 factor model (n = 180).

Item	Factor Loading (FL)			
	F1	F2	F3	F4
19. Nervousness	0.757			
02. Disruption of sleep	0.745			
03. Impact on daily schedule	0.740			
01. Alteration in daily routine	0.735			
14. Sadness	0.721			
18. Frustration	0.666			
10. Outlook on life	0.661			
13. Day-to-day focus	0.660			
15. Mental strain	0.659			
11. Level of stress	0.624			
33. Future outlook	0.568			
29. Change in priorities	0.565			
17. Guilt	0.540			
09. Death of patient	0.530			
20. Impact of illness on family	0.498			
23. Informed about illness	0.361*			
28. Family communication		0.589		
22. Relationship with patient		0.546		
27. Focus of caregiving		0.494		
34. Family support		0.463		
21. Patient’s eating habits		0.407		
12. Spirituality		0.376*		
16. Social support	0.352*	0.373*	0.322*	
06. Financial strain			0.693	
07. Concern about insurance			0.669	
08. Economic future			0.650	
26. Responsibility for patient’s care			0.581	
24. Transportation			0.524	
35. Family interest in caregiving			0.298*	
05. Maintenance of outside activity				0.862
25. Adverse effect of treatment				0.862
32. Management of patient’s pain				0.493
30. Protection of patient				0.450
31. Deterioration of patient				0.440
04. Satisfaction with sexual functioning				0.419
Eigenvalue	7.975	3.307	2.915	1.863
Variance, %	22.785	9.447	8.329	5.323
Cumulative variance	22.785	32.232	40.562	45.885
Removed	12, 16, 23, 35			

*:FL＜0.4

**Table 2:** EFA of 8 factor model (n = 180).

Item	Factor Loading (FL)							
	F1	F2	F3	F4	F5	F6	F7	F8
14	0.835							
15	0.793							
19	0.766							
13	0.737							
18	0.673							
33	0.666							
09	0.624							
11	0.621							
10	0.613							
20	0.580							
17	0.532							
24		0.712						
03		0.780						
01		0.758						
02		0.750						
26		0.518						
29		0.440						
28			0.800					
22			0.760					
21			0.678					
27			0.641					
34			0.583					
16			0.433					
07				0.729				
08				0.692				
06				0.688				
23					0.705			
35					0.608			
25						0.972		
05						0.398*		
31							0.692	
32							0.680	
04							0.385*	0.297
30								0.479
12								0.375*
Eigenvalue	8.44	3.50	2.24	2.17	2.03	1.64	1.40	1.18
Variance, %	24.12	9.99	6.39	6.19	5.80	4.70	3.99	3.36
Cumulative variance	24.12	34.10	40.49	46.68	52.47	57.17	61.16	64.52
Removed	04, 05, 12							

*:FL＜0.4

Both models were tested using CFA. The criteria to assess model fit are illustrated in Table 3. For M2, the CFA revealed a good fit based on the Root mean square error of approximation (RMSEA) criterion [0.058 (90% CI = 0.051-0.065)], CFI (0.96), NNFI (0.96), IFI (0.97), NFI (0.92), and, χ2 (531) = 875.50, *p* < 0.0001. A moderate fit was observed based on the GFI (0.79), but AGFI evidenced a poor fit (values less than 0.90; 0.75). For M1, the CFA revealed close to an adequate fit based on χ2 (522) = 1166.98, *p* < 0.0001. There was a moderate fit based on IFI (0.91), CFI (0.91), and NNFI (0.90), but a poor fit occurred with NFI (0.84), AGFI (0.67), GFI (0.71), and the RMSEA criterion [0.087(90% CI = 0.081-0.093)].

**Table 3:** Goodness-of-fit indices for the two factor models.

Index	4 factor model	8 factor model	Reference value
Goodness of fit index (GFI)	0.71	0.79	＞0.90
GFI adjusted for degrees of freedom (AGFI)	0.67	0.75	＞0.90
Chi-square	1166.98	875.50	χ2/df＜5
Chi-square DF	522	531	
Pr＞Chi-square	＜0.0001	＜0.0001	＜0.05
RMSEA estimate	0.087	0.058	＜0.06
RMSEA 90% lower confidence limit	0.081	0.051	
RMSEA 90% upper confidence limit	0.093	0.065	
Incremental Fit Index (IFI)	0.91	0.97	＞0.95
Bentler’s comparative fit index(CFI)	0.91	0.96	＞0.90
Bentler & Bonett’s non-normed index (NNFI)	0.90	0.96	＞0.95
Bentler & Bonett’s Normed-fit index (NFI)	0.84	0.92	＞0.90

## Discussion

In this study, we evaluated the factor structure of the CQOL-C administered to Chinese mainland caregivers, using both EFA and CFA to assess the hypothesized model developed for the original American sample. A second model that includes eight factors provided a better fit for this Chinese mainland caregiver sample than did the original four factor model.

Different demographic characteristics of caregiver samples and medical characteristics of the patients have been reported. Weitzner et al. [18] assessed two samples at different phases of the illness trajectory (curative or palliative cancer treatment), whereas Weitzner and McMillan [19, 20] studied samples from palliative care settings. A Turkish sample came from a university hospital. A Taiwanese sample hailed from five teaching hospitals in Taiwan, while a French sample came from oncology departments at two French hospitals. In addition, both Weitzner et al. and Weitzner and McMillan restricted the caregivers to those of patients with lung, breast, and prostate cancer. A majority of the Turkish patients had lung cancer (78.1%). French partners had prostate (38%) and breast (22.7%) cancer. Our sample includes caregivers of individuals with various kinds of cancer, which makes our sample largely comparable in demographic and medical characteristics with those assessed in other studies. Research results indicate that the average age of major caregivers on the Chinese mainland is 40, which is much lower than that in the United States (60) [20] and China-Taiwan (60) [25], but only slightly lower than observed for the Turkish (40.19) [22] and French samples (57.6)[24]. Cultural and societal differences might be responsible for this pattern.

Two models were set using EFA. First, we examined the original theorized model, setting a four factor model (Table 1). Overall, four items (12, 16, 23, and 35) had low FLs (< 0.4). The model trends to a single dimension. The first factor, including 16 of the 35 items, also contains 8 of 10 items from the “Burden” subscale (9, 11, 14, 17, 18, 19, 20, and 33) in the original study and 3 of 7 items (1, 3, and 29) from the “Disruptiveness” subscale. In the second factor, 7 items (12, 16, 22, 27, 28, and 34) belong to the third subscale of the original study, “Positive adaption”. In the third factor of our study, items 6, 7, and 8 belonged to the original “Financial concerns” subscale, with the four items from the last factor (4, 30, 32, and 35) not loading onto the original four subscales. Interestingly, we found that the whole model seems to combine the first two subscales of Weitzner’s model, and the other items are all very close to the original English model. In M2 (Table 2), factors with eigenvalues greater than 1.0 were retained. A total of eight factors were extracted. Items 4, 5, and 12 were removed. The results were quite clear: The first four factors include the main items that constitute the original four factors, while the last 4 factors include items that do not load onto these original four factors (see Table 4).

**Table 4:** Comparison of CQOLC, CQOLC-T, CQOLC-M and CQOLC-C.

Original English version (CQOLC)		Simplified Chinese Version (CQOLC-C)		Traditional Chinese Version (CQOLC-M)		Turkish Version (CQOLC-T)	
Subscale	Item	Subscale	Item	Subscale	Item	Subscale	Item
1. Burden, α = 0.89	09	1. Burden, α = 0.88	09*	1. Burden, α = 0.85	09*	1. Psychological distress α = 0.83	09*
	11		11*		11*		11*
	14		14*		14*		14*
	17		17*		17*		18*
	18		18*		18*		19*
	19		19*		19*		20*
	20		20*		20*		25*
	25		33*		31*		31*
	31		10		33*		21
	33		13		13		15
			15		15		
2. Disruptiveness, α = 0.83	01	2. Disruptiveness, α = 0.86	01*	2. Disruptiveness, α = 0.83	01*	2. Disruption on daily life, α = 0.79	01*
	03		03*		03*		03*
	05		24*		21*		05*
	21		26*		24*		29*
	24		29*		26*		02
	26		02		29*		13
	29				02		
					30		
					32		
					35		
3. Positive adaptation, α = 0.73	10	3. Positive adaptation, α = 0.82	16*	3. Social support, α = 0.72	16*	3. Caregiving responsibility, α = 0.73	24*
	12		22*		22*		26*
	16		27*		28*		17
	22		28*		34*		30
	27		34*		23		32
	28		21				33
	34						
4. Financial concern, α = 0.81	06	4. Financial concern, α = 0.81	06*	4. Financial concern, α = 0.80	06*	4. Financial concern, α = 0.77	06*
	07		07*		07*		07*
	08		08*		08*		08*
Other	02	Other	23*	5. Spiritual well-being, α = 0.64	10		
	04		30*		12		
	13		32*				
	15		35*				
	23		25				
	30		31				
	32						
	35						
Overall α	0.9	0.88		0.87		0.88	
Cumulative variance		46.68%		48.15%		40.83%	
Removed items		04, 05, 12		04, 05, 25, 27		04, 10, 12, 16, 22, 23, 27, 28, 34, 35	

*: items which confirm to the original study

According to the CFA results, we choose M2 as the model that best fits a Chinese mainland caregiver sample. Using CFA to compare the 4-factor model from the original English version with M2, the latter is a moderately good fit and superior to M1 according to the standard of a goodness-of-fit index (GFI) of at least 0.90 and an adjusted GFI (AGFI) of > 0.90 adopted by many researchers. To better explain the model and draw comparisons with other studies, we emphasize the first four factors as the main subscales of the CQOLC-C, and consider the other four factors separately.

Table 4 shows the structure of the CQOLC-C and other versions of the scale. Across all studies, only these four versions have been closely examined from a factor structure standpoint. The four subscales of the original English version include 27 items, and the corresponding CQOLC-C subscales include 26 items. The CQOLC-M and CQOLC-T include 28 and 25 items in the first four subscales, respectively. The first subscale is “Burden”. Although in the Turkish version, this subscale is named “Psychological distress”, most of the items were still retained in the CQOLC. Compared with the original structure, the CQOLC-C Burden scale contains items 9, 11, 14, 17, 18, 19, 20, and 33. Items 25 and 31 are not included, all of which belong to the “Other” item category. Items 10, 13, and 15 were added to this subscale. These three items are “outlook on life”, “day-to-day focus”, and “mental strain”, which are all associated with caregivers’ mental stress levels. The second subscale is “Disruption of daily life” for the CQOLC-T, and “Disruptiveness” for the other three versions. The CQOLC-C version contains five items from the original subscale. These are items 1, 3, 24, 26, and 29. Item 5 was removed due to a low FL. Item 2, “Disruption of sleep”, loads on this factor in the Chinese culture. It is also loaded onto this subscale for the other two versions. The third subscale is named “Positive adaptation” for the CQOLC and CQOLC-C, and “Social support” and “Caregiving responsibility” for the other two versions. The CQOLC-C version of the scale includes items 16, 22, 27, 28, 34, and 21. Item 21, “Patient’s eating habits”, assesses whether the caregiver has adapted to changes in the patient’s eating habits, and could therefore quite conceivably belong to this subscale. The fourth subscale is named “Financial concern” for all versions, which each contain items 6, 7, and 8, all of which pertain to financial issues. The CQOLC-M includes a fifth subscale -“spirituality”, which includes items 10 and 12. The English version has the highest Cronbach’s alpha of 0.91, with the CQOLC-C and CQOLC-T both having values of 0.88. The first four factors account for 46.68% of the cumulative variance in CQOLC-C scores, with the entire eight factor scale accounting for 64.52% of the variance.

Three items were removed from the CQOLC-C: Items 4, 5, and 12. Item 4 is “satisfaction with sexual functioning”. In the Chinese culture, sexual life is very private. It is considered rude to talk about this subject in public, and most individuals are unwilling to discuss this topic even for the purposes of scientific research. Such information must be collected in a more indirect way. Item 5, “Maintenance of outside activity”, is difficult to comprehend for people who had no special interests outside of family life. In the Chinese culture, nation is more important than family, which in turn is more important than the individual. In this culture, the importance of family trumps the interests of the individual. When someone develops cancer, their relatives usually try everything to save them. If the patients do not want to be saved, their relatives will attempt to persuade them to “think about your parents or children”. The happy of families is considered more important than individual pain and suffering, and it is considered inappropriate or even selfish to talk about personal activity outside of the family environment in life and death situations. Item 12, “spirituality”, is difficult to explain in Chinese and also difficult for Chinese people to understand. In China, the concept of spirituality may be related to religion. According to research conducted in 1995, 36.09% of Chinese people state that they have no religious affiliation, 28.1% have religious faith, and the remaining 45.81% could not indicate what they believe [30]. Some individuals do not identify with a particular religion but do have faith in God and destiny [31]. Some people believe in more than one religion, and some do not even know what they believe in. This complex belief system derives from a combination of Chinese traditional religions: Confucianism, Buddhism and Taoism, which is further divided into many folk religions and superstitions [32].

Besides culture, age may be another impact factor. In our research, the average age of caregivers was lower than in other samples (40 years old) [19, 23–25]. In China, if the patients are middle aged, their spouses will have the heaviest responsibility to take care of them. If the patients are elderly people, their adult children must take up the responsibility, even giving up work if necessary. This kind of sacrifice is related to the filial piety notion in Chinese culture [33]. So most caregivers are in middle age.

Family structure could be another reason for results differences across studies. The mean age of Chinese caregivers is close to that of the Turkish sample. Both the Chinese mainland and Turkey are developing areas, with a fairly similar standard of living. According to Zeynep [23], the family structures found in Turkey and China are similar. Nevertheless, the population of Chinese young people born after 1980 is much smaller than their elder generations. Most of them are only child from urban families. Grandparents, parents, and a child form a “421” family, which makes it more difficult to take care of elders [34]. However, in recent years, new social problems have arisen. In many rural families, young parents travel a great distance to work in cities. Their children will be left with their grandparents or neighbors, so-called “left-behind children” (58 million in 2008) [35]. When children grow up and move out, their parents usually have retired from work and stay at home alone, so-called “empty nest elderly” (91 million in 2011) [36]. These phenomena have become more and more common in Chinese family, which in turn influences the Chinese social, economic, and medical situation. All in all, the cultural understanding of death and caregiving changes along with societal, economic, technological, and other cultural changes, making it all the more important to refine an existing assessment instrument according to characteristics of the local culture.

There maybe other factors have influence the result of the study such as translation issues. In cultural translation, the differences between original version and translated version is unavoidable. The only thing we can do to minimize the differences is to follow the culture-translation procedure strictly. According to the language experts, our translation is appropriate, accordance with the original version.

QoL of cancer caregivers is multidimensional. This is the basic reason that different versions of this instrument have different factor structures. The strengths of this study lie in the two EFA models trial and the comparison with other versions, which provide a new approach to analyzing the factor structure of a foreign-developed scale. This is the first Chinese mainland study of the CQOLC, which provides a multidimensional assessment of the impact of caregiving on caregiver QoL in the collectivistic Chinese culture. In this study, we recruited a large sample size of caregivers and performed both EFA and CFA using different data sets, which helps assure the statistical accuracy of the results. Further analyses of the reliability and validity of this instrument in the Chinese mainland population will be described in subsequent papers, and we acknowledge that the lack of reliability and validity data renders the present work incomplete.

This study has demonstrated some differences in the factor structure of the CQOLC scale between the Chinese mainland and other samples. As a preliminary study, this research lends only partial support for the relevance and construct validity of the scale for Chinese caregivers. Further work is needed to validate the CQOLC with other well-developed measures with proven cross-cultural validity. The instrument may require further development and modifications before it can be used for routine assessment of Chinese mainland caregivers.
